# Age distribution and gender differences in psychogenic fever patients

**DOI:** 10.1186/1751-0759-3-6

**Published:** 2009-04-21

**Authors:** Yuko Kaneda, Sadatoshi Tsuji, Takakazu Oka

**Affiliations:** 1 Division of Psychosomatic Medicine, Department of Neurology, University of Occupational and Environmental Health, Iseigaoka 1-1, Yahatanishi-ku, Kitakyushu, 807-8555, Japan; 2 Department of Psychosomatic Medicine, Graduate School of Medical Sciences, Kyushu University, 3-1-1 Maidashi, Higashi-ku, Fukuoka 812-8582, Japan

## Abstract

Psychogenic fever is one of the most common psychosomatic diseases. In Japan, psychogenic fever has generally been reported to occur in adolescents, with a peak seen at age 13. However, in our department we have encountered many adults with psychogenic fever. Therefore, we investigated all outpatients who visited the Psychosomatic Department of the University of Occupational and Environmental Health between April 2003 and March 2007. Of the 2705 outpatients that were seen, 55 patients (2.0%) were diagnosed with psychogenic fever. The patients ranged in age from 11 to 82 years old, with a mean age of 33.6 ± 17.9 (mean ± SD) years. In addition to the adolescents, many of the patients were in their 20 s and 30 s, and the male:female ratio was 1:2.2. This study suggests that psychogenic fever commonly occurs not only in adolescents but also in adults.

## Findings

Psychogenic fever is one of the most common psychosomatic diseases. A person is diagnosed with psychogenic fever when their core body temperature (Tc) exceeds the normal range, not due to an organic cause but because of psychological stress. However, in spite of numerous case reports on psychogenic fever, epidemiological studies have been limited. In 2007, we reviewed 62 articles on psychogenic fever that were published in Japan [1]. In all, there were 195 cases reported, with a mean patient age of 14.3 years and a male:female ratio of 1:1.2 [1]. Psychogenic fever was primarily seen in adolescent patients, and only 14% of cases occurred in patients 20 years old or older. However, in our department we have encountered many adult patients who experienced low-grade fever during psychologically stressful situations without any abnormal findings [2,3]. In our previous report we found that children exhibited higher body temperatures than adults, and we hypothesized that pediatricians were more easily alerted to stress-induced hyperthermic responses than internists. This being the case, it is understandable that many of the reports on psychogenic fever have come from pediatricians. In the present study, we investigated the prevalence of psychogenic fever in adults.

Of those outpatients who visited the Psychosomatic Medicine Division, Department of Neurology, University of Occupational and Environmental Health for the first time between April 2003 and March 2007, there were 2705 new patients. Sixty-one patients were referred to our department due to the occurrence of a fever of unknown origin with possible involvement of psychosocial factors. In the present study, we made a diagnosis of psychogenic fever when the patient fulfilled the following criteria: (1) axillary temperature above 37.0°C, (2) no inflammatory signs or endocrinological disease to account for the high Tc, and (3) the fever developed during a psychosocially stressful situation. The stressful situation could be an emotional event such as an argument, or in some cases the fever was reproduced by a stressful interview. Cases were excluded if the fever turned out to be factitious or was associated with chronic fatigue syndrome. Of the 61 referred cases, 4 were excluded due to chronic fatigue syndrome and 2 were excluded because the fever was found to be factitious after the patients were hospitalized.

**Figure 1:**
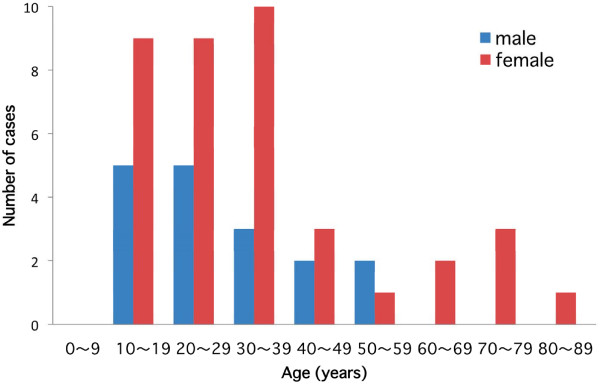
**Age distribution of psychogenic fever patients**.

The 55 patients diagnosed with psychogenic fever corresponded to 2.0% of all new patients who visited our department over the 4-year period. There were 17 males (30.9%) and 38 females (69.1%), and the male:female ratio was 1:2.2. The patients ranged in age from 11 to 82 years, with a mean age of 33.6 ± 17.9 years. Patients in age groups 10–19 and 20–29 years old were the most numerous, with 14 patients in each group (25.5%). Thirteen patients were 30–39 years old (23.6%). The number of cases was small for all age groups 40 years old or above, with at most only 1–5 cases for each group. The mean age was 30.1 ± 13.1 years for all the male patients and 35.2 ± 19.6 years for all the female patients, and there was no significant difference between males and females (Fig. 1).

In this study, 41 of 55 patients (74.5%) were 20 years old or more, suggesting that psychogenic fever frequently occurs not only in adolescents but also in adults. This result is in contrast to our previous review paper that reported that psychogenic fever patients were most numerous at age 13, with a mean age of 14.3 years. Although the reason for the age distribution in the present study is unclear, it is possible that the results were affected by the age distribution of patients who visited our department, since the number of outpatients 15 years or younger was only 3.6% of all patients seen during the investigation period. Most previous case reports on psychogenic fever have come from pediatricians [1], and the difference in our results might be a reflection of the small number of young patients treated at our department.

The male:female ratio in this study was 1:2.2, whereas in our previous review study it was 1:1.2 [1]. It is possible that this gender difference was related to the age of the patients since in the previous study the male:female ratio of patients less than 11 years old was 1.3:1, and that of patients 11 years and older was 1:1.4 [1]. As there were no patients who were less than 11 years old in the present study, the female predominance may be reasonable. Female predominance is seen in other psychosomatic diseases such as irritable bowel syndrome [4] and tension-type headache [5]. Considering that the male:female ratio changed around 11 years old, sex hormones might also play a role in the development of psychogenic fever.

In the present study, 75% of the psychogenic fever patients were 20 years old or older. Nozu *et al. *investigated 83 patients complaining of a fever that persisted for more than 2 weeks without any abnormal findings, and concluded that the cause of fever in 40 cases (48%) seemed to be psychogenic [6]. The mean age of patients in that study was 29 years. Taken together, the findings suggest that there may be many more adult psychogenic fever patients than previously believed based on the few cases reported. When adult patients present with fever of unknown cause and no abnormal findings, physicians should consider psychogenic fever.

## Authors' contributions

YK analyzed the data and drafted the manuscript. ST supervised the study. TO helped to analyze the data and write the manuscript. All authors have read and approved the final manuscript.
